# Electrochemical Cell Chips Based on Functionalized Nanometals

**DOI:** 10.3389/fchem.2021.671922

**Published:** 2021-05-06

**Authors:** Waleed Ahmed El-Said, Jinho Yoon, Sang-Nam Lee, Jeong-Woo Choi

**Affiliations:** ^1^Department of Chemical and Biomolecular Engineering, Sogang University, Seoul, South Korea; ^2^Department of Chemistry, Faculty of Science, Assiut University, Assiut, Egypt; ^3^Department of Chemistry and Chemical Biology, Rutgers, The State University of New Jersey, Piscataway, NJ, United States; ^4^Uniance Gene Inc., Seoul, South Korea

**Keywords:** cell-based chip, nanometal, electrochemical detection, biosensor, nanoarray

## Abstract

The electrochemical technique is one of the most accurate, rapid, and sensitive analytical assays, which becomes promising techniques for biological assays at a single-cell scale. Nanometals have been widely used for modification of the traditional electrodes to develop highly sensitive electrochemical cell chips. The electrochemical cell chips based on the nanostructured surface have been used as label-free, simple, and non-destructive techniques for *in vitro* monitoring of the effects of different anticancer drugs at the cellular level. Here, we will provide the recent progress in fabrication of nanopatterned surface and cell-based nanoarray, and discuss their applications based on electrochemical techniques such as detection of cellular states and chemicals, and non-destructive monitoring of stem cell differentiation.

## Introduction

Monitoring the different chemicals' levels plays a vital role in predicting the changes from normal physiology to disease physiology. However, the biological heterogeneity of the living systems showed the unique challenge to monitor the chemical levels. Cell-based sensors have been widely used as attractive assays for previous investigations (Yea et al., 2008). Electrochemical cell-based chips have been widely studied for (1) investigating the fundamental cellular functions including motility, proliferation, differentiation, and apoptosis, (2) evaluation of the effects of different anticancer drugs, (3) drug discovery, and (4) cell-external stimuli interactions (El-Said et al., 2009). Recently, nanomaterials have attracted the massive attention because of the excellent electrochemical behaviors that could provide the promising immobilization matrices and improve the electrochemical conductivity and, hence, the development of high sensitivity electrochemical cell-based chips could be achieved (Shin et al., 2020).

**Figure 1:**
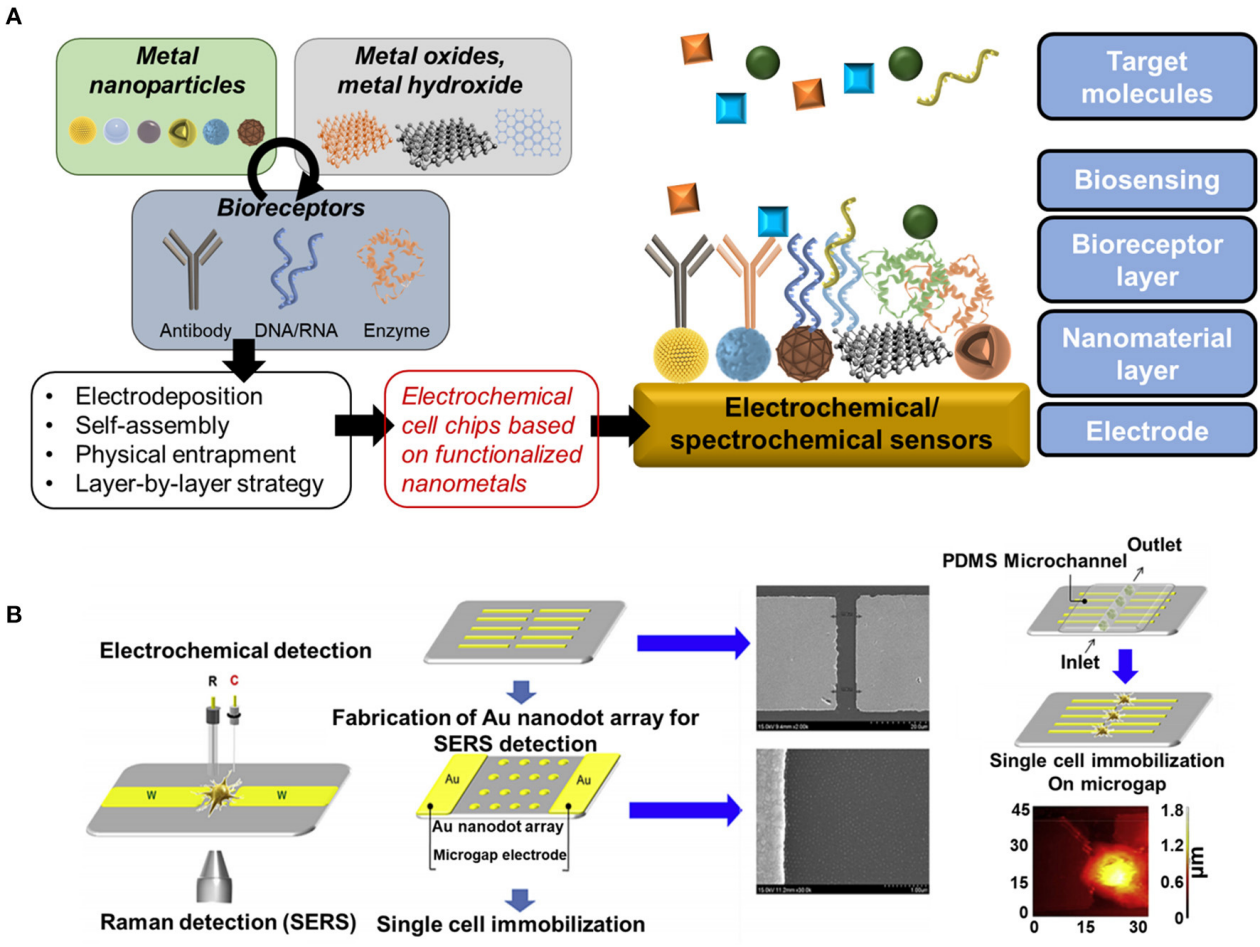
**(A)** Schematic diagram of main components and representative strategies for development of nanomaterials-based electrochemical biosensor and **(B)** Schematic diagram of the immobilization of a single cell on a microgap between pairs of Au microelectrodes. Reproduced with permission from El-Said et al., 2015b (Copyright ©2015 Elsevier).

In addition, a cell-based nanoarray chip has been studied a lot recently for monitoring stem cell differentiation (Zheng et al., 2017; Lee et al., 2020). Particularly, a conductive nanomaterial-based nanoarray chip has a huge potential for non-destructive electrochemical cell monitoring because of its advantages, including the excellent conductivity and improvement of cell alignment (Figure 1A) (Cui et al., 2017; Ino et al., 2018; Lee J.-H. et al., 2018). The non-destructive monitoring is one of the most importantly required characteristics in stem cell therapy for direct use of differentiated cells to the clinical treatment after monitoring in non-destructive manner. For this, several techniques such as electrochemical deposition and laser interference lithography (LIL) have been utilized to develop the novel cell-based nanoarray chip for cell differentiation monitoring (El-Said et al., 2015a).

This mini-review focuses on some of the electrochemical cell chips based on the nanostructured surface and their applications, which provides a broad overview of electrochemical cell chips to be applied for biomedical applications, including the evaluation of drug effect or toxicity and *in vitro* cell differentiation monitoring.

## Nanopatterned Surface to Detect Cellular State and Chemicals Based on Electrochemical Techniques

### Metal Nanoparticles-Based Electrochemical and Spectrochemical Sensors

A cell-based chip consisting of arginylglycylaspartic acid-methionylalaninylproline-cysteine (RGD-MAP-C peptide)@gold nanoparticles (Au NPs)/ITO was used to evaluate the proliferation of undifferentiated human neural stem cells (HB1.F3) and the effects of doxorubicin on HB1.F3 cells (Kim et al., 2013). Furthermore, a single cell-based chip was reported, a pair of Au microelectrodes with a micrometer-sized gap between the electrodes. A hexagonal array of Au nanodots were deposed inside the micro-gap. This cell-based chip was used to study the intracellular and extracellular redox state of PC12 cells based on a spectroelectrochemical tool that combined surface-enhanced Raman scattering (SERS) and linear sweep voltammetry techniques simultaneously (Figure 1B). This approach could be used as an effective research tool to analyze cellular processes at a single cell level (El-Said et al., 2015b).

Furthermore, the Au-Pt NPs were deposited onto Au multi-electrode array and used as a cell-based chip. Pt was introduced to GNPs to improve the electrical properties of the modified electrode. The Au-Pt NPs exhibited moderate neuronal cell affinity comparable to GNPs. We have checked the efficacy of Au-Pt NPs by recording noise levels and spike signals and by observing cultures of primary neuronal cells. The sensor was used to record the neuronal signals from primarily cultured neuron cells (Kim et al., 2016).

Besides, monitoring the critical biomarkers in the human body represented an essential basis for health assessment, pharmaceutical guidance, surgical intervention protocols, and postsurgical monitoring (Khan et al., 2019; Li et al., 2020; Patella et al., 2021). Nowadays, there is a high interest in developing efficient strategies to diagnose cancer and fight against it. The early detection of neoplastic tumors is an important issue because the treatment in the early stages is more efficient. Monitoring cancer biomarkers is one of the most widely used methods for cancer diagnosis. Fibroblast growth factors (FGFs) and their tyrosine kinase receptors (FGFRs) are relevant in various biological processes. The fibroblast growth factor receptor 4 (FGFR4) alterations play an essential role in developing cancer in the breast, ovarian, prostate, colon, rhabdomyosarcoma, pancreatic, and gastric, hepatocellular, and pituitary adenocarcinomas. The FGFR4 overexpression is associated with metastasis and the late stages of cancer. Torrente-RodrõÂguez et al. (2017) have presented an electrochemical immunosensor for determining fibroblast growth factor receptor 4 (FGFR4) biomarker in different cancer cell lysates. This biosensor was composed of carbon screen-printed electrodes (SPCEs) modified with a specific capture antibody, and detection was performed by using the amperometric technique, which showed a LOD of 48.2 pg mL^−1^ (Torrente-RodrõÂguez et al., 2017).

The level of nitric oxide (NO) in physiological environments plays a vital role in several biological processes such as neurotransmission, immune responses, cardiovascular systems, angiogenesis, microcirculation, etc. Changes in the NO levels are associated with inflammation, neurovirulence, and cancer progression. Thus, the development of a real-time NO sensor could be of great importance for human health. However, there are many challenges for NO sensing due to the very low NO concentration in physiological environments and the interference with other chemicals, including glucose, nitrites, and uric acid. Li et al. have fabricated a flexible and biodegradable electrochemical NO sensor (Li et al., 2020). The sensor was based on an ultrathin Au nanomembrane with a thickness of 32 nm as a working electrode. The sensor was used for monitoring NO released in cultured cells and organs over several days with a detection limit of 3.97 nmol. The work may provide essential diagnostic and therapeutic information for health assessment, treatment optimization, and postsurgical monitoring. Furthermore, they have reported its application for monitoring NO in the joint cavity for 5 days. Thus, this sensor could be used as a NO sensor in physiological conditions and as a promising candidate for diagnostic and therapeutic information uses (Li et al., 2020).

Moreover, fabrication of porous Au film modified electrodes was reported to enhance the electrochemical conductivity and developing highly sensitive cell-based chip for rapid cancer detection (El-Said et al., 2010) electrochemical biosensors (Fouad and El-Said, 2016; Bollella et al., 2019; Jo and Shin, 2020). The unique electrochemical, optical, and biocompatible properties of the noble metals NPs besides their ability to form in several shapes with a larger surface area represented one of the best candidates to enhance the efficiency of electrochemical/spectrochemical-based biosensors. However, their highly-cost effect could limit their commercialized applications.

Increasing the generation of reactive oxygen species (ROS) leads to most inflammatory diseases' pathogenesis besides its association with aging and chronic inflammation. Hydrogen peroxide (H_2_O_2_) received considerable attention due to the vital role of H_2_O_2_ in directing the leukocytes to the injury site (Patella et al., 2021). Thus, the accurate monitoring of H_2_O_2_ derived from living cells is of critical significance to control the progression of congenital diseases such as cancer and damage to DNA. Several non-enzymatic electrochemical sensors based on nanometals modified electrodes were reported. Abdelwahab and Shim have constructed silver NPs (Ag NPs) modified oxidized poly-2,2:5,2-terthiophene-3-p-benzoic acid/multi-wall carbon nanotube (OxpTTBA/MWCNTs) electrode for sensing H_2_O_2_ (Abdelwahab and Shim, 2014). Furthermore, Pt NPs modified nanoporous Au electrode was used for electrochemical determination of H_2_O_2_ (Yin et al., 2014). The use of nanoporous Au increases the surface area and promotes electron-transfer reactions. Sophia et al., have used Au NPs stabilized in polyvinylpyrrolidone (PVP) modified GCE as an H_2_O_2_ sensor (Sophia and Muralidharan, 2015). Moreover, Au with different morphologies including rod, sphere, and cubic surrounded with palladium (Pd) as supra-nanoparticles were used as an H_2_O_2_ sensor. These structures provide a higher active surface area than that of the Au@Pd continuous shell nanoparticles (Huang et al., 2015). Yuan et al. (2017) have fabricated monolayer graphene/Au NPs modified GCE based on CVD-generated graphene to avoid the complicated polymer transfer, or cleaning processes. This sensor is superior to GCEs modified with AuNPs on reduced graphene oxide (Yuan et al., 2017). Furthermore, graphene/Au nanoparticle-paper electrode was applied as simple and sensitive H_2_O_2_ sensor (Liu et al., 2019). Also, pencil graphite electrode (PGE) was modified with a poly(2-aminophenylbenzimidazole)/Au NPs (P2AB/Au NPs) and used as an H_2_O_2_ sensor that could detected H_2_O_2_ with no interference by ascorbic acid, dopamine, uric acid, or glucose (Teker et al., 2019). Additionally, Patella et al. (2021) have used rGO/Au NPs/ITO/PET electrode as an H_2_O_2_ sensor for direct detecting of H_2_O_2_ that released by cells in culture supernatants (Patella et al., 2021).

### Metal Oxides, Metal Hydroxide and Their Composites-Based Electrochemical Sensors

H_2_O_2_ monitoring has been successfully done using a wide variety of nanomaterials, including inorganic hydroxides, metal oxides, and metals (Zhang et al., 2018). An innovative and successful photoelectrochemical sensor was designed for H_2_O_2_ analysis using platinum and nickel hydroxyl-oxide double-layer optimized n-silicon wafer electrodes. Correspondingly, the nanoporous layer of BiVO_4_ was developed on the surface of fluorine-doped tin oxide glass for the photoelectrochemical inspection of H_2_O_2_ to use a simple electro-deposition process. Suitable photo-catalytic, electrochemical, and photoelectrochemical sensing applications were shown by ZnO NPs. A hybrid structure changed electrode for selective photoelectrochemical detection of H_2_O_2_ derived from living Hela cells following stimulus with N-formylmethionyl-leucyl-phenylalanine was recorded for the development of C-dot (polydopamine derived carbon dots) wrapped ZnO NPs (fMLP). The ZnO NPs demonstrated good biocompatibility with the cell lines of Huh7. In the ZnO NPs coated by dopamine-derived C-dots, there were numerous catalytic active sites, broad surface defects, high electrical conductivity, and effective separation potential of photo-induced electrons and holes. These findings suggested that H_2_O_2_ released from living Hela cells has been successfully controlled photo-electrochemically by changed the electrode (Lee et al., 2019).

On the other hand, an electrochemical biosensor for detecting eosinophil cationic protein (ECP) as an asthma biomarker in the cell culture (Lee C.-Y. et al., 2018). The sensor was based on a screen-printed electrode (SPE) in the presence of heparin-modified Au@Fe_3_O_4_ NPs. The uses of magnetic NPs play a vital role in the sensor fabrication, in which the Au@Fe_3_O_4_ NPs were attached with the electrode's surface based on applying an external magnetic field. The sensing principle was based on the specific affinity of heparin toward ECP. Thus, the heparin-modified magnetic NPs were mixed with the ECP sample and apply an external magnetic field that results in the aggregation of the NPs onto the SPE. This sensor showed a LOD of about 0.30 nM with a good recovery in a cell culture medium. The ECP in the sample was adsorbed onto the dispersed heparin-modified Au@Fe_3_O_4_ NPs, which aggregated onto the SPE's surface.

The level of nitric oxide (NO) in physiological environments plays a vital role in several biological processes such as neurotransmission, immune responses, cardiovascular systems, angiogenesis, microcirculation, etc. Changes in the NO levels are associated with inflammation, neurovirulence, and cancer progression. Thus, the development of a real-time NO sensor could be of great importance for human health. However, there are many challenges for NO sensing due to the very low NO concentration in physiological environments and the interference with other chemicals, including glucose, nitrites, and uric acid. Li et al. have fabricated a flexible and biodegradable electrochemical NO sensor (Li et al., 2020). The sensor was based on an ultrathin Au nanomembrane with a thickness of 32 nm as a working electrode. The sensor was used for monitoring NO released in cultured cells and organs over several days with a detection limit of 3.97 nmol. The work may provide essential diagnostic and therapeutic information for health assessment, treatment optimization, and postsurgical monitoring. Furthermore, they have reported its application for monitoring NO in the joint cavity for 5 days. Thus, this sensor could be used as a NO sensor in physiological conditions and as a promising candidate for diagnostic and therapeutic information uses (Li et al., 2020).

## Nanoarray to Monitor Stem Cell Differentiation Based on Electrochemical Technique

**Figure 2:**
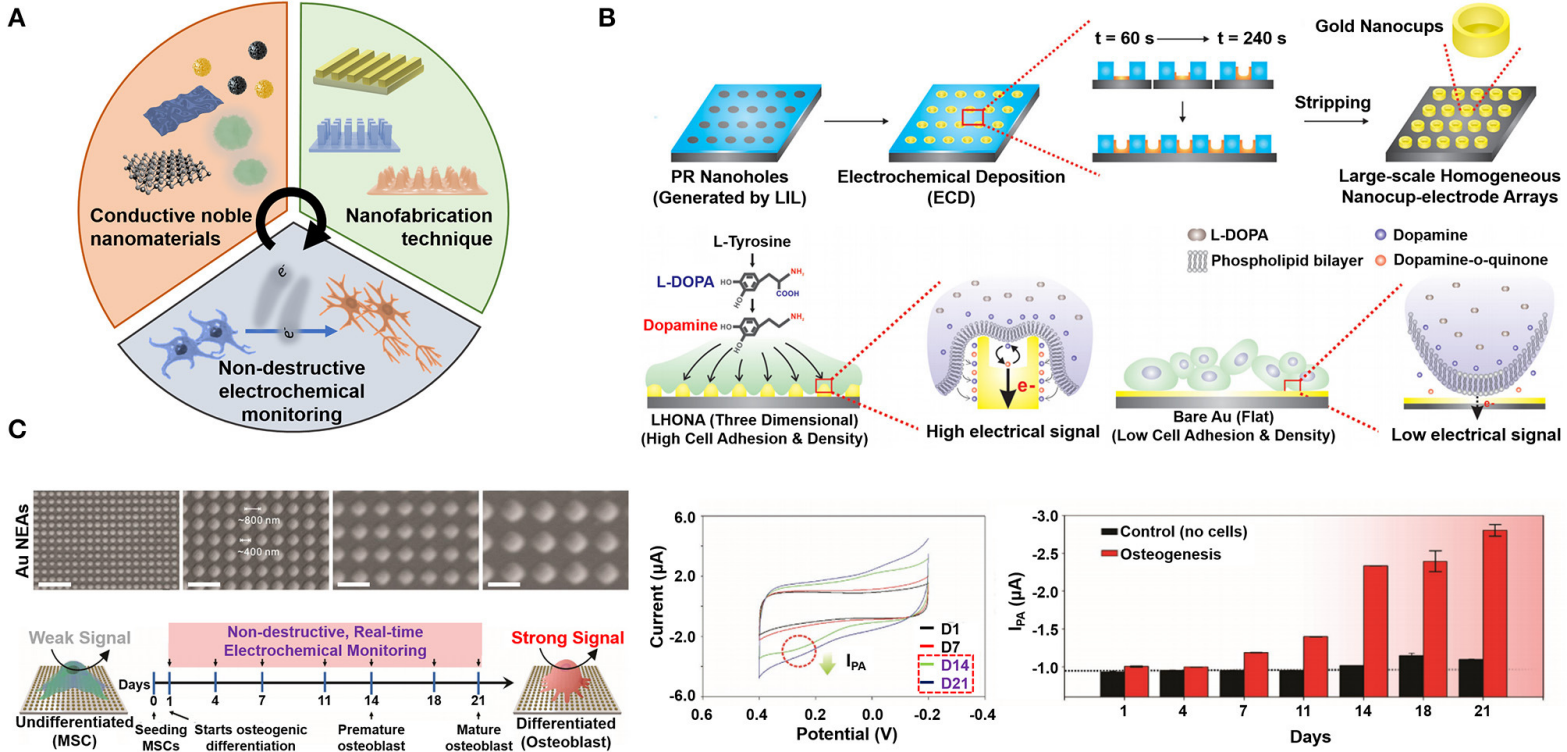
**(A)** Schematic diagram for properties and components of metallic nanoarray-mediated non-destructive monitoring of stem cell differentiation based on electrochemical technique. **(B)** Schematic diagram of LHONA fabrication on the ITO electrode using the LIL and electrochemical deposition method, and scheme for effective interaction between cells and the surface of nanocup-like structure compared to flat (2D) surface. Reproduced with permission from Kim et al. (2015b). Copyright (2015) John Wiley and Sons. **(C)** Scanning electron microscopy (SEM) images of NEAs after physical vapor deposition of the gold based on the pitch sizes in 400, 800, 1,200 and 1,600 nm (40,000× magnification), schematic diagram of electrochemical signal changes by osteogenic differentiation based on ALP expression, and cyclic voltammograms and calculated current values of non-destructive monitoring of osteogenic differentiation from 1 to 21 days. Reproduced with permission from Lee J.-H. et al. (2018). Copyright (2018) John Wiley and Sons.

The *in vitro* stem cell differentiation is one of the important issues in biomedical fields to be used for stem cell therapy and regenerative medicine (Álvarez-Errico et al., 2015; Nemati et al., 2019). However, conventional monitoring methods such as fluorescent monitoring require the cell fixation process which inevitably destruct the differentiated cells for investigation that hinders the direct utilization of differentiated cells for biomedical application. The electrochemical technique provides the promising strategy for stem cell differentiation monitoring without cell destruction steps including the cell fixation (Low et al., 2017). Therefore, non-destructive monitoring can be achieved that is one of the most importantly required property to be applied for stem cell therapy through direct use of differentiated cells to the therapy after monitoring of its cellular states in non-destructive manner. In addition, the electrochemical technique suggests the novel approach to fabricate the nanomaterial-based unique structural nanoarray to be used for cell monitoring. From these points of views, the electrochemical technique has been utilized to develop the cell-based nanoarray and has been studied to propose the effective non-destructive stem cell differentiation monitoring tool by combination with conductive nanoarrays (Figure 2A).

Among various electrochemical methods, the electrochemical deposition method has been particularly used to fabricate the unique structural nanoarrays on the conductive electrode (Li et al., 2018). By electrochemical deposition, the unique nanoarrays can be fabricated on the substrate easily and rapidly by controlling of parameters such as applied potential intensity, applied time, and concentration of deposited metal solution. Various reported nanoarrays have been used for stem cell differentiation monitoring. For example, the gold nanostar arrays were developed by electrochemical deposition to be used for the *in vitro* monitoring of neural stem cell differentiation (El-Said et al., 2015a). In another research, the spiky structural nanoelectrode array was developed by electrochemical deposition to control and monitor the neuronal differentiation of mesenchymal stem cells (MSCs) (Poudineh et al., 2018). However, the electrochemical deposition has a critical limitation that metal deposited area or nanostructure-formed region could not be controlled because deposition was occurred on the whole surface area randomly.

To overcome this limitation, several novel techniques have been utilized to develop the electrochemical monitorable nanoarray composed of state-of-the-art nanostructures. For example, the combinatorial graphene-based hybrid arrays with different geometries were developed by using the polydimethylsiloxane (PDMS) stamping method (Kim et al., 2015a). This stamping method provided the method for fabrication of various structural graphene-based hybrid arrays such as the line, grid and dot arrays, which structures were evaluated to find the optimal structure for differentiation of MSCs. In addition, using the stamping method, the metallic nanopattern array was developed through the combined use of stamping method and nano-transfer printing technique (Hwang et al., 2019). For achieving this goal, authors prepared the nanopattern template using the stamping method, and then, metal ions were deposited on the prepared nanopattern template. Next, deposited metal nanolayer was transferred to the adhesive substrate at nanometer scale to form the metallic nanopattern array formation. This study showed that the stamping method can be utilized for not only graphene-based nanomaterials patterning, but also nanopatterning of noble metals. Besides, recently the electrochemical micropyramid nanoarrays were proposed for *in situ* monitoring of dopamine from cells (Senel et al., 2020). To develop this novel micropyramid nanoarrays, the PMDS micropyramid was prepared using the silicone MPatch micropyramid template, and then, the chrome and gold were deposited on the micropyramid by the magnetron sputtering process. Developed metallic micropyramid nanoarrays showed the excellent sensing performance of dopamine released from neuroblastoma cells with the detection limit of 0.50 ± 0.08 nM and a wide linear range of 0.01–500 μM, which suggested the novel conceptual approach to develop the electrochemical monitoring platform for stem cell differentiation. In addition to these examples, several methods have been used to fabricate the metallic nanoarray such as the electrochemical nanopattern formation (ENF) process, photolithography and electroplating (Hwang et al., 2013; Shin et al., 2019). Although these studies showed the appealing strategies to develop the novel nanoarray, their complex or many steps for fabrication hinder their application for stem cell monitoring efficiently.

Among various novel techniques, the facile LIL technique provides the powerful strategy to develop the multifunctional electrochemical cell-based nanoarray chip in relatively simple fabrication manner. As a mask-less lithographic technique, the LIL is more convenient than the other lithography techniques to fabricate millions of nanostructures using the photosensitive materials like the photoresist molecules (Lu and Lipson, 2010). In addition, by combining with electrochemical deposition, each fabricated nanostructure can be carefully modified with conductive metallic nanomaterials like the gold and graphene (Yang et al., 2019). Moreover, the LIL, that can make nanostructures uniformly on the substrate at the nanometer scale, can generate various nanostructure arrays including the line, grid, hole and orthogonal structures by controlling of some parameters such as the laser reflection angles, the laser exposure and developing times, and types of photoresist molecules. Using this facile technique, several cell-based nanoarray chips were reported for monitoring of non-destructive stem cell differentiation based on electrochemical technique. For instance, the large-scale homogeneous nanocup-electrode array (LHONA) was developed using the LIL and electrochemical deposition method for enhancement of cell-to-nanostructure interactions and cell signal monitoring (Kim et al., 2015b). The LHONA is the cup-like nanostructure which was fabricated by electrochemical deposition of gold inside the photoresist-mediated LIL patterned array. By electrochemical deposition, the deposition rate of the gold was controlled to form the cup-like nanostructure which was impossible to be achieved by the other deposition methods including physical vapor deposition and electron-beam deposition. The fabricated LHONA showed the extended surface area of the gold for inducing the effective interaction between the nanocup structures and stem cells compared to the other nanoarrays (Figure 2B). Moreover, because of its extended metallic conductive surface area of nanoarrays, the LHONA successfully conducted *in situ* detection of the dopamine at 10 × 10^−6^ M concentration level, and the electrochemical signals from dopaminergic differentiated cells were detected with high sensitivity (*I*_*pc*_: 0.13 uA). This study verified that cell-based nanoarray chip could provide the powerful strategy for monitoring of non-destructive stem cell differentiation based on electrochemical technique.

In addition to this study, the graphene-gold hybrid nanoelectrode arrays (NEAs) were developed for not only non-destructive monitoring, but also for enhancing the stem cell differentiation (Figure 2C) (Lee J.-H. et al., 2018). Here, the LIL and physical vapor deposition of the gold were utilized to make the graphene-gold hybrid NEAs. After fabrication of gold NEAs, reduced graphene oxide covered the gold NEAs for enhancing the electron transfer rate and biocompatibility. By controlling of the laser reflection angles of the LIL, authors found the optimized condition of nanoarrays for osteogenic differentiation of MSCs [The pitch size (PS) of NEAs: 400 nm]. Using this NEAs composed of the 400 nm PS, MSCs were differentiated into osteogenic cells effectively. Besides, using the alkaline phosphatase (ALP) which is overexpressed from cells during osteogenic differentiation, the non-destructive electrochemical monitoring of stem cell differentiation was conducted through the redox reaction between expressed ALP and additionally added P-aminophenyl phosphate (PAPP). From non-destructive monitoring results, the electrochemical signals were apparently detected from the cells which was differentiated in day 14 and 21. Still, several issues related to conductive nanoarray-based electrochemical monitoring should be solved, such as the reduction of complex fabrication steps and diversification of nanomaterials that can be used, by interdisciplinary approaches for commercial application. In addition, further studies are required, such as the development of nanoarrays using recently reported novel nanomaterials and optimization of conditions of nanoarrays to be used for specific cell differentiation control more effectively. As discussed here, various cell-based nanoarrays have been developed using the electrochemical technique and these nanoarrays provided the appealing strategy for non-destructive electrochemical monitoring of stem cell differentiation.

## Conclusion and Future Perspective

The electrochemical cell-based chips are *in-vitro* microfabricated devices mimicking the cell functions, widely used as promising, rapid, precise, and non-destructive tools. The electrochemical cell-based chips were widely used for drug screening, studying the cellular functions, studying cell-to-cell interaction, and detection of small biomolecules, including DNA, enzymes, and hormones. Particularly, the nanopatterned surfaces for detecting cellular state and chemicals, cell-based nanoarray chips have been studied hugely for non-destructive electrochemical cell differentiation monitoring, which is essentially required for stem cell therapy, using several novel techniques such as LIL and electrochemical deposition. Developed cell-based nanoarray chips showed excellent biocompatibility and highly sensitive monitoring property for stem cell differentiation. Still, further studies are required to solve several issues and to develop the effective nanoarrays for specific cell differentiation control by interdisciplinary approaches for commercial application. Nevertheless, researches discussed in this mini-review provides interdisciplinary knowledge and new approaches to develop the electrochemical cell chips for numerous biomedical applications such as evaluation of drug effect/toxicity and *in vitro* cell differentiation monitoring. Furthermore, cell-based chips would contribute in several fields such as (i) accurate medical diagnosis, (ii) disease diagnosis, (iii) quality assurance of stem cell-based products, (iv) generate more human-like biology, and (v) more human-relevant, including personalized medicine (i.e., precision medicine).

## Author Contributions

All authors listed have made a substantial, direct and intellectual contribution to the work, and approved it for publication.

## Conflict of Interest

S-NL was employed by Uniance Gene Inc. The remaining authors declare that the research was conducted in the absence of any commercial or financial relationships that could be construed as a potential conflict of interest.
